# CT colonography has advantages over colonoscopy for size measurement of colorectal polyps

**DOI:** 10.1007/s11604-024-01625-0

**Published:** 2024-06-29

**Authors:** Daisuke Tsurumaru, Yusuke Nishimuta, Katsuya Nanjo, Satohiro Kai, Mitsutoshi Miyasaka, Toshio Muraki, Kousei Ishigami

**Affiliations:** 1 Department of Clinical Radiology, Graduate School of Medical Sciences, Kyushu University, 3-1-1 Maidashi, Higashi-Ku, Fukuoka, Japan; 2 Department of Gastrointestinal Endoscopy, National Hospital Organization, Kyushu Cancer Center, Fukuoka city, Japan

**Keywords:** Colorectal polyp, CT colonography, Colonoscopy, Colorectal cancer

## Abstract

**Purpose:**

The aim of this study was to compare the accuracy of colonoscopy (CS) and CT colonography (CTC) in the measurement of colorectal polyps using pathological size as a reference.

**Materials and methods:**

The analysis included 61 colorectal polyps in 28 patients who underwent preoperative CTC at our institution. All polyps were endoscopically resected. Polyp sizes were measured by CS and CTC. Endoscopic polyp size was extracted from endoscopy records written by one of two endoscopists (A with 11 and B with 6 years of endoscopic experience, respectively), who estimated the size visually/categorically without any measuring devices. After matching the location, the polyp size was measured on CTC using manual three-dimensional (3D) measurement on a workstation. The sizes of resected polyps were also measured after pathological inspection. Differences of the polyp size between CTC and histology, and between CS and histology were compared using paired t tests. Differences in measurement between the two endoscopists were also analyzed.

**Results:**

The mean diameters of polyps measured using CS, CTC, and pathology were 10.5 mm, 9.2 mm, and 8.4 mm, respectively. There was a significant correlation between CS and pathology, as well as between CTC and pathology (both *P* < 0.0001). The correlation coefficient for CS (r = 0.86) was lower than that for CTC (r = 0.96). The correlations between CS and pathology for endoscopists A and B were 0.90 and 0.89, respectively.

**Conclusion:**

Measurements of polyp size using CTC were closer to the pathological measurements compared to those by CS, which exhibited greater variability. This suggests that CTC may be more suitable for polyp size measurements in the clinical setting if patients undergo CTC concurrently with colonoscopy.

## Introduction

Colorectal polyps are relatively common growths on the mucosal surface of the colon or rectum and are generally treated by endoscopic resection. The size of the polyps is an important determinant of treatment and surveillance, because it can predict their future behavior [1–5]. Polyp size ≥ 10 mm and high-grade dysplasia have been shown to be associated with colorectal cancer mortality in patients screened by colonoscopy [2]. In another study, colorectal cancer risk was found to be higher for individuals with adenomas ≥ 20 mm in diameter compared to the general populations [1]. Therefore, the precise assessment of colorectal polyp size is of paramount clinical importance. However, colorectal polyps are typically measured via visual estimation by endoscopists, introducing variability associated with the endoscopist’s skill and experience, as well as with the endoscopic conditions, such as the distance between the subject and the endoscope.

Computed tomography colonography (CTC) has emerged as an effective modality for colorectal examination, and even a potentially viable alternative to colonoscopy (CS), and has been widely adopted for both preoperative assessment and colorectal cancer screening. The reported polyp detection sensitivity of CTC for lesions measuring 6 mm or larger is approximately 88–90%, with a specificity of 92–93% [6, 7]. Importantly, CTC not only aids in polyp detection but also enables accurate size measurement due to its digital nature, surpassing the precision achievable through endoscopic visual estimation. The aim of this study was to compare the accuracy of CS and CTC in the measurement of colorectal polyps, with pathological size serving as a reference.

## Materials and methods

### Study participants

**Fig. 1:**
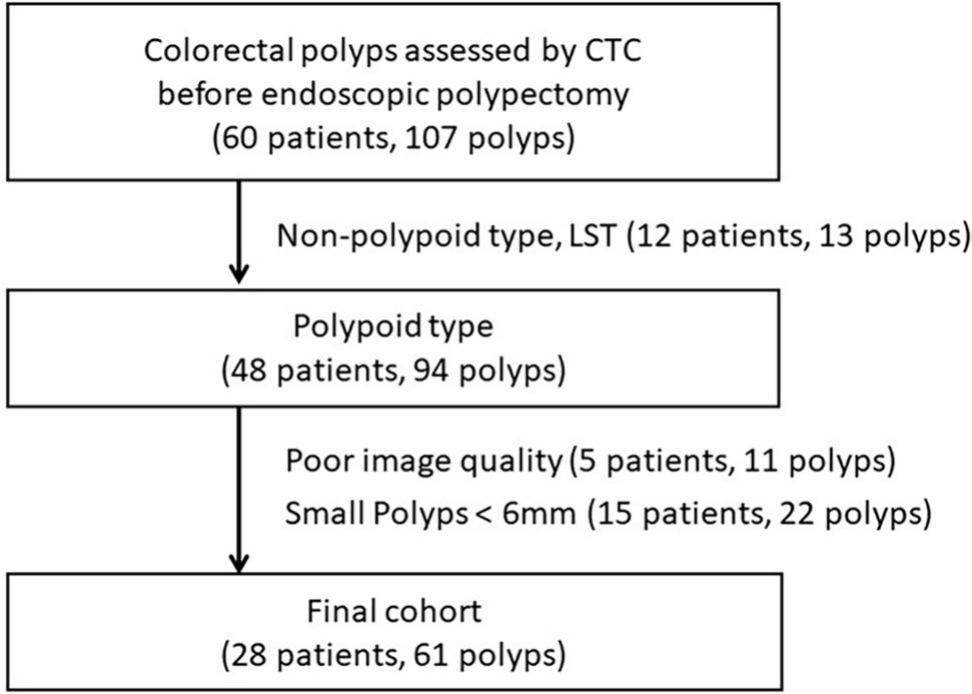
Flowchart of the cohort

This was a retrospective, single-center, observational study approved by our institutional review board, which waived the requirement for informed consent. Data on 107 colorectal polyps from 61consecutive patients seen between January 2015 and August 2018 were included; all polyps were assessed by CTC before endoscopic polypectomy. All patients were suspected of having colorectal cancerous polyps, including cancer in adenomas. Consequently, these polyps included lesions suspected to be cancerous, as well as other polyps associated with those patients. Small lesions less than 6 mm in diameter (*n* = 22), non-polypoid lesions including laterally spreading tumors (*n* = 13), and polyps with poor CTC image quality mainly due to poor colonic distension or a large amount of residual fluid (*n* = 11) were excluded. Polyps smaller than 6 mm were excluded because they are generally not assessed by CTC due to their extremely low malignant potential. Our final study cohort consisted of 28 patients with 61 colorectal polyps (Fig. 1). Baseline data for the patients and polyps are shown in Table 1.

**Table 1:** Demographics

Gender, male/female		26/2
Age (y, median)		39–80 (63)
Location	Cecum	2
	Ascending colon	7
	Transverse colon	10
	Descending colon	6
	Sigmoid colon	24
	Rectum	12
Gross type	Ip/Isp/Is	11/26/24
Histology	Adenoma	54
	Adenocarcinoma in adenoma	3
	Adenocarcinoma	2
	Hyperplastic	2
Treatment	Endoscopic resection	61

### Initial colonoscopy and polyp size measurement

For each patient, CS was performed using a bowel preparation method utilizing polyethylene glycol. Two board-certified endoscopists (A and B) who had 11 and 6 years of experience in screening and therapeutic endoscopy performed the endoscopic procedures by random assignment. They detected lesions and measured the size (the longest diameter) visually without using any measurement devices, and these values were recorded in the endoscopic reports.

### CT colonography procedures

CTC imaging was performed after CS on the same day. Before CT scanning, a single balloon tube was inserted into the rectum by the transanal route, and colonic insufflation with carbon dioxide using a CO2 injector (PROTOCO2L; Bracco, Princeton, NJ) was performed. The CT scan was performed using a 320-slice CT (Aquilion One; Canon Medical Systems) with the following parameters: 120 kV, 100–300 mA, beam collimation 1 mm, slice thickness 1 mm, reconstruction interval 1 mm, and pitch 65. Contrast-enhanced CT using an iodine agent (Iopamiron 370; Bayer Health Care) was performed for 15 patients who were suspected to have cancer to assess liver metastasis. Patients were positioned in the supine position for the arterial and portal phases and the prone position for the delayed phase. No tagged agent was used.

### CT colonography analysis

**Fig. 2:**
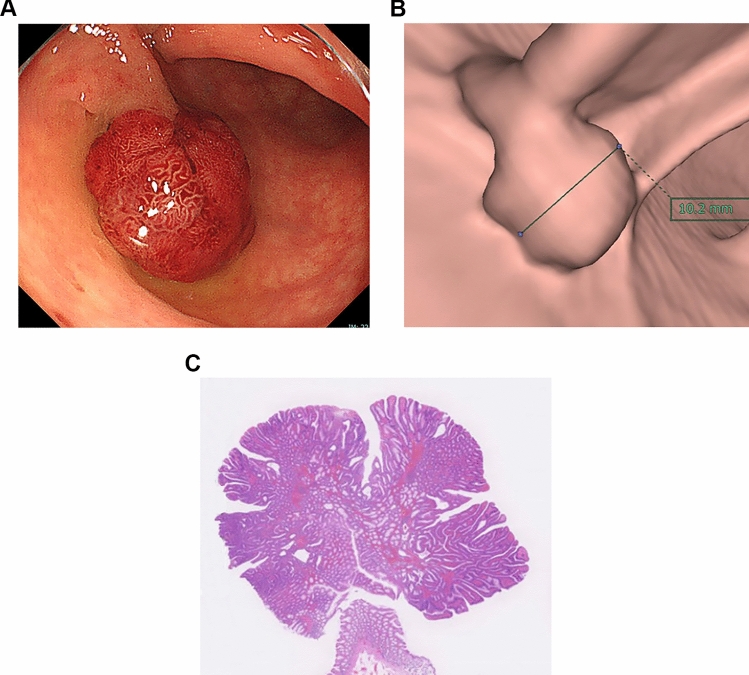
**a** CS shows a pedunculated polyp in the sigmoid colon; the longest diameter estimated by Endoscopist A was 20 mm. **b** CTC shows the same polyp, measured as 10.2 mm. **c** Microscopic picture after polypectomy shows tubulevillous adenoma, measured as 10 mm

The CTC data sets were loaded onto a workstation (Synapse Vincent, Fujifilm Medical). All CTC images were reviewed in consensus by two board-certified radiologists who had 13 and 7 years of experience interpreting CTC. The colorectal polyps were identified using the initial CS records as a reference, and then the maximum diameter of the polyps was measured on virtual endoscopy mode from the same viewpoint as the respective CS (Fig. 2).

### Endoscopic procedure and pathology

All 61 colonoscopic procedures were performed with an interval of less than 6 months after CS and CTC. After identification of the polyps with the data provided by these modalities, all polyps were resected by conventional endoscopic mucosal resection or cold snare polypectomy. No complications associated with the procedure were reported. Resected polyps were immediately fixed in formalin solution. After pathological inspection, the long diameter of the largest pathological section of each polyp was directly measured using a ruler.

### Statistical analysis

The polyp size (longest diameter) was compared between CS and pathology and CTC and pathology using Pearson’s correlation coefficient with the pathological polyp size as a reference. Results of the former test were subdivided by endoscopist. Differences with *p*-values < 0.05 were accepted as significant. To assess variability between those modalities and pathological measurement, data were displayed in Bland–Altman plots. Statistical analyses were performed using JMP pro 16 software (SAS, Cary, NC).

## Results

**Fig. 3:**
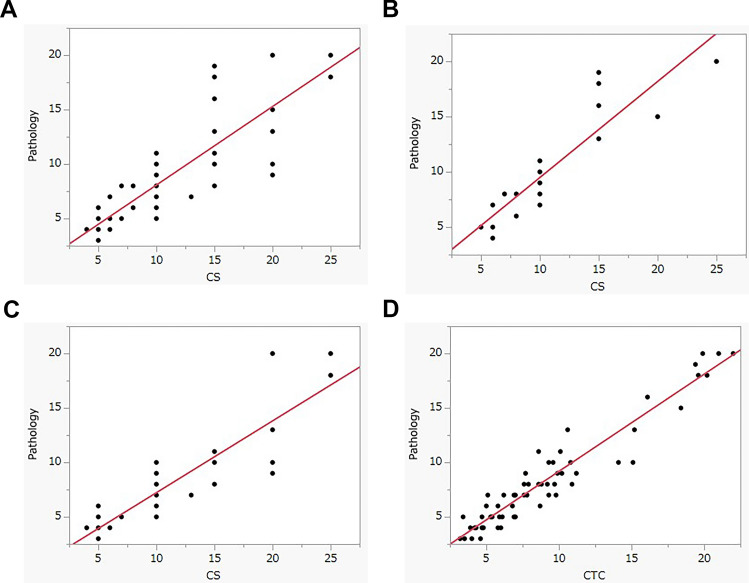
**a** The positive correlation between CS and pathology for colorectal polyp size measurement (coefficient of 0.86). **b** and **c** Correlation between endoscopic sizes for endoscopists A and B, respectively: Measurements showed a positive correlation with coefficients of 0.90 and 0.89, respectively. **d** Correlation between CTC and pathological size: There was significant correlation with a coefficient of 0.96

**Fig. 4:**
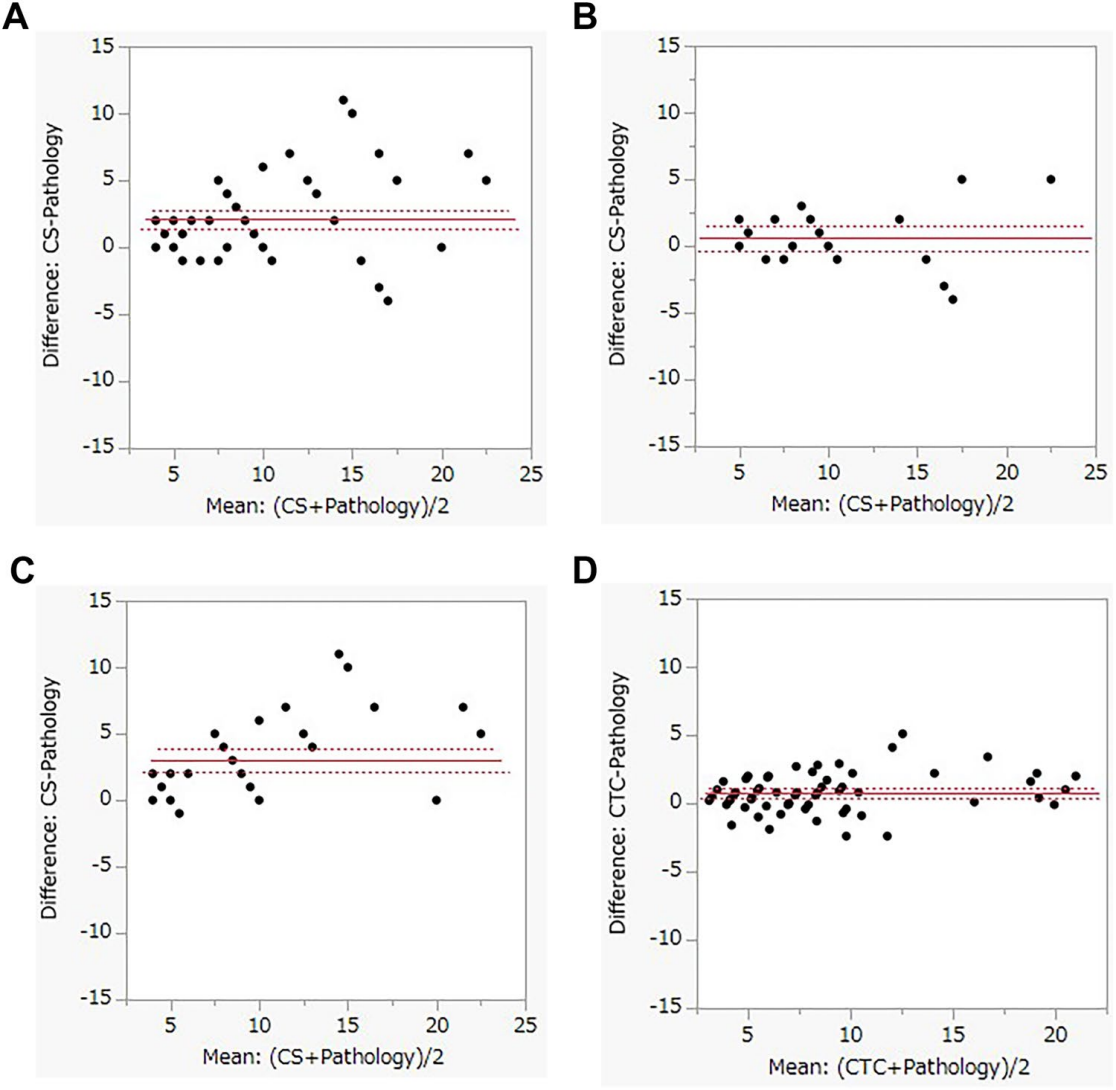
**a** Bland–Altman plot of polyp measurements exhibiting the large variability in the difference between measurements by CS and pathology. **b** and **c** Bland–Altman plot of polyp measurements performed with CS (endoscopist A and B) versus pathology: Endoscopist B exhibited greater variability in the measurements compared to endoscopist A. **d** Bland–Altman plot of polyp measurements performed with CTC versus pathology: The difference between the measurements of CTC and pathology exhibited less variability than that between CS and pathology

The mean diameters of polyps measured visually using CS, CTC, and pathology were 10.5 mm, 9.2 mm, and 8.4 mm, respectively. There was a significant correlation between CS and pathology, as well as between CTC and pathology (both *P* < 0.0001). However, the correlation coefficient for the former comparison (r = 0.86) was lower than that for the latter (r = 0.96). In terms of the correlation between CS and pathology, the correlation coefficients for endoscopists A and B were 0.90 and 0.89, respectively. However, the difference from the pathologic value for endoscopist A (mean, 0.5 mm) was lower than that for endoscopist B (mean, 3.0 mm). Additionally, note that the CS measurements were rounded to convenient numbers such as 5 mm or 10 mm (Table 2, Fig. 3). The corresponding Bland–Altman plots are presented in Fig. 4. The difference in measurement values between CTC and pathology was smaller than that between CS and pathology.

**Table 2:** Results of polyp size measurement

	Diameter (mean ± SD, mm)	Difference from pathology (mean ± SD, mm)
Colonoscopy (total, *n* = 61)	10.5 ± 0.7	2.1 ± 2.8
Endoscopist A (*n* = 23)	10.4 ± 1.1	0.5 ± 2.2
Endoscopist B (*n* = 38)	10.6 ± 5.8	3.0 ± 2.7
CT colonography (*n* = 61)	9.2 ± 0.6	0.7 ± 1.5
Pathology (*n* = 61)	8.4 ± 0.6	

SD = standard deviation

## Discussion

Our retrospective single-center observational study aimed to assess the accuracy of polyp size measurements obtained via CS and CTC, in comparison with pathological assessments. The precise measurement of colorectal polyp size holds significant clinical importance due to the association of size and growth rate with the risk of malignancy and treatment strategy.

Our analysis showed that both CS estimations and CTC measurements were significantly correlated with the pathological measurements, affirming their utility in providing a good sense of polyp size, notwithstanding the difference in the respective sizes: 10.5 mm (mean diameter) by visual measurement using CS versus 9.2 mm by CTC analysis. The results also showed that the correlation coefficient between CTC and pathology was notably higher than that observed for CS and pathology, and the standard deviation of CTC was lower than that of CS, indicating that CTC shows less variability than CS.

Several studies have attempted to determine the accuracy of the various devices used for polyp size measurement during CS [8–11]. The standard practice for size determination is visual estimation by the endoscopist during CS, which is a source of bias for several reasons. When size categories are present, endoscopists tend to assign some diameters more frequently than others, leading to overestimation and underestimation of true size [8–17]. In this study, as well as in previous reports, there was variability among observers, and there was a tendency to use convenient round numbers such as 5 mm and 10 mm. Image distortion can also occur through the endoscope lens, making polyp size determination dependent on the angle of view. The commonly used guide of opening biopsy forceps is also unreliable [8]. These issues can lead to both intra- and inter-observer variability in polyp size measurements [9, 18–20]. In agreement with our present results, Kim et al. found that endoscopists with less experience may exhibit greater measurement variability compared to experts [20]. Physiochemically, post-fixation measurements are smaller than those made immediately after polypectomy [8, 12].

There have been several studies comparing the accuracies of polyp size measurement using CTC and CS [21–25]. These data suggest that polyp size measurements determined by CTC are typically intermediate to those made at pathologic evaluation and CS and may be closer to the in vivo size [18]. Specifically, the polyp sizes estimated using CS were on average approximately 1–3 mm larger than those made by CTC, which is close to the findings of this study [18, 21]. Based on the results of a previous retrospective cohort study, the determination of references for polyp size measurement is controversial and challenging [2]. In this regard, CTC serves as a highly objective and reproducible measurement method with potentially minimal variability. In a clinical setting, the indication for endoscopic treatment and the malignant risk of colorectal polyps depends on their size. Since there is a margin of error in endoscopic size measurements, our present results indicate that the CTC measurements should be adopted, if available, when accurate size measurement is necessary.

Polyp size measurements by CTC can be affected by factors during acquisition and display of the images as well as factors during the process of obtaining the measurements. Display factors include the use of 2D multiplanar versus 3D endoluminal images, the choice of rendering thresholds and window settings, the quality of colon segmentation, and the effects of high-attenuation endoluminal contrast agents [18]. Several reports have found endoluminal 3D measurements to be the most accurate, with a tendency for 2D measurements to underestimate the maximal transverse diameter [24, 25]. In this study, we adopted polyp measurements on virtual endoscopy using the same viewpoint as for CS, an approach that we considered intuitive, straightforward, and reasonable.

Several limitations should be acknowledged in this study. First, our study sample size was relatively small because we assessed data from only a single institution, potentially limiting the generalizability of our findings. Additionally, in endoscopy, colorectal polyps were only measured by one observer because we adopted the polyp size recorded in the endoscopic report—i.e., the estimation made by the endoscopist in an actual clinical setting. On the other hand, colorectal polyp sizes measured using a 3D virtual endoscopy were determined by consensus of two radiologists. Finally, although the polyp size measurements using CS and CTC were both based on in vivo information within the same individual, the condition of the colon, including the insufflation volume and colonic distention, may have differed. Additionally, the time interval between the initial endoscopy and the endoscopic treatment might have affected the results. These differences could have introduced measurement bias. Despite these limitations, this study makes a novel contribution through its direct comparison of CS, CTC, and pathological measurements within the same set of polyps.

In conclusion, measurements of polyp size using CTC were closer to the pathological measurements than those determined by CS, which exhibited greater variability. This suggests that CTC may be more suitable for polyp size measurements in the clinical setting if patients undergo CTC concurrently with colonoscopy. Further research with larger sample sizes and a prospective design is warranted to validate these findings and determine their clinical implications.
